# Altered Calcium and Vitamin D Homeostasis in First-Time Calcium Kidney Stone-Formers

**DOI:** 10.1371/journal.pone.0137350

**Published:** 2015-09-02

**Authors:** Hemamalini Ketha, Ravinder J. Singh, Stefan K. Grebe, Eric J. Bergstralh, Andrew D. Rule, John C. Lieske, Rajiv Kumar

**Affiliations:** 1 Division of Nephrology and Hypertension, Mayo Clinic, Rochester, Minnesota, United States of America; 2 Department of Medicine, Mayo Clinic, Rochester, Minnesota, United States of America; 3 Department of Laboratory Medicine and Pathology, Mayo Clinic, Rochester, Minnesota, United States of America; 4 Department of Health Sciences Research, Mayo Clinic, Rochester, Minnesota, United States of America; 5 Department of Biochemistry and Molecular Biology, Mayo Clinic, Rochester, Minnesota, United States of America; Tel Aviv Sourasky Medical Center, ISRAEL

## Abstract

**Background:**

Elevated serum 1,25-dihydroxyvitamin D (1,25(OH)_2_D) concentrations have been reported among cohorts of recurrent calcium (Ca) kidney stone-formers and implicated in the pathogenesis of hypercalciuria. Variations in Ca and vitamin D metabolism, and excretion of urinary solutes among first-time male and female Ca stone-formers in the community, however, have not been defined.

**Methods:**

In a 4-year community-based study we measured serum Ca, phosphorus (P), 25-hydroxyvitamin D (25(OH)D), 1,25(OH)_2_D, 24,25-dihydroxyvitamin D (24,25(OH)_2_D), parathyroid hormone (PTH), and fibroblast growth factor-23 (FGF-23) concentrations in first-time Ca stone-formers and age- and gender frequency-matched controls.

**Results:**

Serum Ca and 1,25(OH)_2_D were increased in Ca stone-formers compared to controls (P = 0.01 and P = 0.001). Stone-formers had a lower serum 24,25(OH)_2_D/25(OH)D ratio compared to controls (P = 0.008). Serum PTH and FGF-23 concentrations were similar in the groups. Urine Ca excretion was similar in the two groups (P = 0.82). In controls, positive associations between serum 25(OH)D and 24,25(OH)_2_D, FGF-23 and fractional phosphate excretion, and negative associations between serum Ca and PTH, and FGF-23 and 1,25(OH)_2_D were observed. In SF associations between FGF-23 and fractional phosphate excretion, and FGF-23 and 1,25(OH)_2_D, were not observed. 1,25(OH)_2_D concentrations associated more weakly with FGF-23 in SF compared with C (P <0.05).

**Conclusions:**

Quantitative differences in serum Ca and 1,25(OH)_2_D and reductions in 24-hydroxylation of vitamin D metabolites are present in first-time SF and might contribute to first-time stone risk.

## Introduction

Urinary stone disease is an increasingly common and recurrent metabolic disorder with an estimated lifetime risk in the United States of 6–12%, and a recurrence rate of up to 50%. [1–4]. Both dietary patterns and genetic factors influence urinary Ca excretion and are important in the pathogenesis of kidney stones [5–10]. Earlier reports from tertiary referral clinics demonstrated elevated serum concentrations of the active metabolite of vitamin D, 1,25(OH)_2_D [11, 12], among patients with hypercalciuric nephrolithiasis with or without primary hyperparathyroidism [13–17]. Increased 1,25(OH)_2_D levels should stimulate the intestinal hyperabsorption of Ca and contribute to the hypercalciuria observed in such patients. The precise cause of elevated 1,25(OH)_2_D concentrations in hypercalciuric stone-formers is uncertain, although some have suggested that low normal serum phosphorus concentrations may drive increased 1,25(OH)_2_D synthesis [17]. A recent report of 356 male incident stone formers compared plasma concentrations of 25-hydroxyvitamin D, 24,25-dihydroxyvitamin D, parathyroid hormone, fibroblast growth factor 23, calcium, phosphate, and creatinine with those found in control subjects [18]. Small and statistically non-significant differences in 1,25(OH)_2_D and FGF23 levels were found in cases compared to controls. However, after adjusting for multiple covariates, the odds ratios of incident symptomatic kidney stones in the highest compared with lowest quartiles were 1.73 (P for trend 0.01) for 1,25(OH)2D and 1.45 (P for trend 0.03) for FGF23. Urinary solute concentrations were not examined.

Mutations and polymorphisms of specific genes have been associated with an increased risk of kidney stones [19–26]. Recently, infants [19, 20, 27] and adults [19, 28] with elevated serum 1,25(OH)_2_D concentrations, hypercalcemia, hypercalciuria and nephrolithiasis have been found to have inactivating mutations of the 24-hydroxylase cytochrome P450 (*CYP24A1*) gene, the product of which degrades 25(OH)D and 1,25(OH)_2_D to less active metabolites, 24,25(OH)_2_D and 1,24,25-trihydroxyvitamin D, respectively, in target organs such as the intestine and kidney [12, 29]. In this study we asked whether there was evidence for an alteration in mineral metabolism and reduced 24-hydroxylase activity (measured as the ratio of 24,25(OH)_2_D/25(OH)D) among patients with their first kidney stone, the vast majority (>90%) of which are calcium oxalate and/or calcium phosphate. We now show that serum Ca and 1,25(OH)_2_D are higher in a community-based cohort of first-time Ca stone-formers compared to a group of age- and gender-matched normal subjects. Furthermore, subjects with Ca kidney stones have lower 24,25(OH)_2_D/25(OH)D ratios suggesting the presence of reduced 24-hydroxylase activity. We show positive associations between FGF-23 and fractional phosphate excretion in control subjects that are absent in stone-formers. 1,25(OH)_2_D concentrations associated more weakly with FGF-23 in stone-formers compared with controls.

## Materials and Methods

### Stone former cohort and matched controls

The Mayo Clinic Institutional Review Board approved this study (IRB 12–004641). Subjects provided written consent to participate in the study. Documentation of consent was included in the subject’s medical record. The consent form, and procedure for consent were approved by the Mayo Clinic Institutional Review Board. Patients from the 9 county region of Southeast Minnesota, who were seen since 1/1/2009 by a medical provider for their first kidney stone, were invited to participate in the study. A review of medical records confirmed that patients had experienced their first symptomatic kidney stone episode. Controls with no history or kidney stones (frequency matched to the age and gender of enrolled stone-formers) were concurrently recruited from the same local general population using flyers and mail-outs.

### Laboratory studies

Serum creatinine, Ca and P were measured using a Roche Cobas C311 autoanalyzer. Intact PTH was measured using a two-site chemiluminescent assay (Roche), and serum 25(OH)D, 1,25(OH)_2_D and 24,25(OH)_2_D were measured by mass spectrometry [30–32]. Intact full-length FGF-23 was measured using an enzyme linked immunoassay (Kainos). Stone analysis was performed using infra-red spectroscopy in the Mayo Medical Laboratories. Urinary excretion of solutes was analyzed with methods routinely in use at the Mayo Medical Laboratories.

Dietary intake was assessed using a Food Frequency Questionnaire [33].

### Statistical analysis

Univariable comparison of controls to stone-formers was done using the two-sample t-test. Within stone-formers and controls, multivariable linear regression analysis was used to assess associations between pairs of laboratory values (Y,X) while adjusting for age, sex and other factors. Similar models were fit pooling all subjects and included a term for stone-former status and its interaction with X. Statistical analyses were performed on JMP version 10.0.0 (SAS Inc., North Carolina, USA) statistical software.

## Results

Information from 153 first time stone formers was available for analysis. Analysis of stone material by infrared spectroscopy was available in 94 stone-formers revealing Ca oxalate in 73 (78%), hydroxyapatite in 17 (18%), uric acid in 3 and cystine in one (Ca stones confirmed by IR analysis = 90). Dual energy CT scans on 3 additional patients without stone analysis confirmed the presence of non-uric acid stones. Patients with the uric acid stones (n = 3) and cystine stone (n = 1) were not considered in the analysis. Thus, there were 93 (90 by direct IR analysis and 3 by dual energy CT) confirmed Ca stone formers (CSF, 45 males and 48 females (n = 93); age: 48±15 years) and 201 age- and gender frequency-matched controls (95 males, 106 females; age: 45±15 years). For purposes of additional analysis, the remaining 56 stone-formers were assumed to have Ca stones. Thus, there were 149 (93 confirmed + 56 presumed = 149) first-time Ca stone-formers (77 males and 72 females; age: 46±15 years) in the total Ca stone former group (TSF). The results are presented separately for each of the two groups group of first-time kidney stone formers.

As shown in Table 1 compared to controls Ca stone-formers in both the confirmed (CSF) and TSF groups had higher serum Ca (P = 0.03 (CSF); P = 0.01 (TSF)), similar serum P (P = 0.87 (CSF); P = 0.72 (TSF)), higher 1,25(OH)_2_D (P = 0.0004 (CSF); P = 0.001 (TSF)), lower 24,25(OH)_2_D/25(OH)D ratios (P = 0.014 (CSF); P = 0.008 (TSF)), similar serum PTH (P = 0.18 (CSF); P = 0.09 (TSF)) and similar FGF-23 (P = 0.6 (CSF); P = 0.28 (TSF)). Serum 25(OH)D and 24,25(OH)_2_D concentrations were similar in stone-formers vs. controls (P = 0.1 and 0.84 (CSF); P = 0.14 and 0.59 (TSF), respectively).

**Table 1:** Serum analytes and hormones (PTH, 25(OH)D, 1,25(OH)_2_D, 24,25(OH)_2_D and FGF-23) and urine analytes in calcium stone-formers and controls. All values are represented as mean ± standard error. CSF = confirmed; TSF = total (confirmed plus presumed calcium SF).

Biochemical Parameter	Controls (N = 201)	Confirmed calcium stone-formers, CSF (N = 93)	P	Total stone-formers, TSF (N = 149)	P
**Serum chemistries**					
**Calcium, mg/dL**	9.2 ± 0.1	9.4 ± 0.1	**0.03**	9.4 ± 0.04	**0.010**
**Phosphorus, mg/dL**	3.4 ± 0.01	3.3 ± 0.06	0.865	3.4 ± 0.01	0.722
**PTH, pg/mL**	38.8 ± 1.2	41.9 ± 1.8	0.15	42.1 ± 1.5	0.096
**25(OH)D, ng/mL**	32.1 ± 0.7	34.4 ± 1.2	0.105	33.8 ± 0.9	0.136
**1,25(OH)**_**2**_ **D, pg/mL**	38.1 ± 0.9	43.8 ± 1.3	**0.004**	43.7 ± 1.1	**0.001**
**24,25(OH)**_**2**_ **D, ng/mL**	3.2 ± 0.1	3.1 ± 0.2	0.844	3.1 ± 0.1	0.591
**24,25(OH)**_**2**_ **D/25(OH)D**	0.097 ± 0.03	0.088 ± 0.003	**0**.**0143**	0.087 ± 0.03	**0.008**
**FGF23 pg/mL**	59.2 ± 1.6	60.4 ± 2.2	0.656	62.2 ± 2.4	0.278
**Uric Acid, mg/dL**	4.9 ± 0.1	5.2 ± 0.11	**0.023**	5.4 ± 0.1	**0.003**
**Creatinine, mg/dL**	0.82 ± 0.01	0.81 ± 0.01	0.591	0.84 ± 0.2	0.437
**24 hour urine chemistries**					
**pH**	6.2 ± 0.04	6.1 ± 0.05	**0.031**	6.1 ± 0.04	**0.012**
**Volume (mL)**	1794.2 ± 57.1	1659.1 ± 82.5	0.09	1597.3 ± 61.5	**0.015**
**Osmolality**	648.9 ± 17.6	672.3 ± 28.1	0.48	693.6 ± 21.7	0.110
**Sodium, mmol**	131.3 ± 67.3	121.8 ± 6.89	0.259	124.4 ± 67.3	0.344
**Potassium, mg**	53.2 ± 2.4	45.91 ± 2.7	**0.044**	45.2 ± 2.0	**0.014**
**Calcium, mg**	222.3 ± 10.3	225.8 ± 14.4	0.85	225.8 ± 11.6	0.820
**Magnesium, mg**	127.0 ± 4.5	112.33 ± 7.18	**0.042**	113.4 ± 5.3	**0.040**
**Chloride, mmol**	121.0 ± 65.82	112.0 ± 6.6	0.267	113.9 ± 64.9	**0.032**
**Phosphate, mg**	647.7 ± 21.8	645.68 ± 37.97	0.96	651.8 ± 29.2	0.910
**Sulfate, mmol**	21.5 ± 14.8	17.71 ± 2.5	0.169	18.5 ± 16.9	0.133
**Citrate, mg**	596.3 ± 24.7	517.5 ± 31.22	**0.049**	504.0 ± 23.6	**0.010**
**Oxalate, mmol**	0.27 ± 0.1	0.27 ± 0.01	0.998	0.27 ± 0.19	0.950
**Uric Acid, mg**	424.7 ± 12.9	358.74 ± 17.3	**0.0026**	383.2 ± 14.8	**0.036**
**Creatinine Clearance**	92.1 ± 2.5	91.92 ± 4.9	0.978	90.2 ± 45.1	0.668
**% FECa**	1.89 ± 0.07	1.97 ± 0.1	0.542	2.04 ± 0.10	0.218
**% FEPi**	14.89 ± 0.36	15.29 ± 0.6	0.562	15.84 ± 0.57	0.143

Urinary pH, volume, and potassium, magnesium, chloride and citrate excretion were lower in stone formers compared to controls (see Table 1 for P values). Twenty four hour urinary calcium excretion was similar. FECa and FEPi were similar in the two groups.

The results of age- and gender-adjusted multivariable analyses are shown in Table 2. In both controls and stone-formers, serum Ca concentrations were inversely related (negative slope, β) to serum PTH concentrations. The relationship was not significantly different between groups (P = 0.655 for interaction). In controls, serum P concentrations were inversely related to PTH concentrations even after adjusting for 1,25(OH)_2_D concentrations. This relationship was not observed in stone-formers. However, the difference between the two groups was not statistically significant. In controls, 1,25(OH)_2_D concentrations and FGF-23 concentrations were inversely related even after correcting for serum PTH, serum Ca and serum P, while in stone-formers this relationship was not observed. The difference in the associations between the two groups was statistically significant (Table 2, P = 0.05 for interaction). In controls, FE P and FGF-23 concentrations were directly related even after correcting for serum PTH and serum 1,25(OH)_2_D, while in stone-formers this relationship was not observed. The difference between the associations in the two groups was not statistically significant (Table 2, P = 0.136 for interaction). We examined the relationship of other P regulating hormones to FE P in controls and stone-formers. In controls, FE P and 1,25(OH)_2_D concentrations were not related, even after correcting for serum FGF-23, PTH and Ca, while in stone-formers there was a negative relationship between 1,25(OH)_2_D and FE P. The difference in the associations between the two groups was statistically significant (Table 2, P = 0.017 for interaction).

**Table 2:** Multivariable regression analysis of dependent variable (Y) on independent variable (X). Columns include adjusting variables (in addition to age and sex), parameter or slope estimate (ß), standard error (SE), and P value (test of slope = 0) for analyses done within stone former and control groups, Also given is the P value for the interaction term (test for common slopes) between X and stone group from similar models pooling stone-formers and controls. CSF = confirmed Ca stone formers; TSF = total stone formers (confirmed plus presumed).

Dependent (Y) Variable	Independent (X) variable	Adjusted Variable	C, N = 201,ß (SE), P	CSF, N = 93,ß (SE), P	P, Interaction Term	TSF, N = 149, ß (SE), P	P, Interaction
S Ca	PTH		-0.006 (0.002), **0.036**	-0.006 (0.003), **0.017**	0.66	-0.006 (0.003), **0.017**	0.66
S Ca	PTH	1,25(OH)_2_D	-0.0005 (0.002), **0.04**	-0.006 (0.003), **0.018**	0.94	-0.006 (0.003), **0.018**	0.87
S Ca	1,25(OH)_2_D	PTH	-0.00085 (0.03), 0.81	-0.001 (0.003), 0.76	0.91	-0.001 (0.003), 0.76	0.986
S P	PTH		-0.092 (0.03), 0.09	-0.089 (0.07), 0.17	0.77	-0.002 (0.002), 0.315	0.226
S P	PTH	1,25(OH)_2_D	-0.113 (0.05), **0.03**	-0.09 (0.06), 0.14	0.78	-0.002 (0.002), 0.316	0.240
S P	1,25(OH)_2_D	PTH	0.113 (0.05), **0.03**	0.086 (0.07), 0.20	0.19	0.0001 (0.003), 0.968	0.18
1,25(OH)_2_D	PTH		0.101 (0.05), 0.06	0.85 (0.07), 0.21	0.74	0.053 (0.052), 0.31	0.96
1,25(OH)_2_D	PTH	FGF-23	-0.163 (0.04), **<0.001**	-0.035 (0.061), 0.56	**0.050**	0.06 (0.05), 0.32	0.73
1,25(OH)_2_D	PTH	FGF-23, S Ca	-0.168 (0.04), **<0.001**	-0.05 (0.06), 0.46	0.084	0.05 (0.05), 0.33	0.73
1,25(OH)_2_D	PTH	FGF-23, S Ca, S P	-0.169 (0.04), **<0.001**	-0.04 (0.06), 0.47	0.08	0.05 (0.05), 0.34	0.765
1,25(OH)_2_D	FGF-23		-0.160 (0.04), **<0.001**	-0.04 (0.06), 0.48	0.08	-0.057 (0.034), 0.16	**0.050**
1,25(OH)_2_D	FGF-23	PTH	-0.168 (0.04), **<0.001**	-0.05 (0.06), 0.38	0.51	-0.05 (0.037), 0.11	**0.045**
1,25(OH)_2_D	FGF-23	PTH, S Ca	-0.169 (0.04), **<0.001**	-0.05 (0.06), 0.42	0.655	-0.05 (0.037), 0.11	**0.046**
1,25(OH)_2_D	FGF-23	PTH, S Ca, S P	-0.162 (0.04), **<0.001**	-0.05 (0.06), 0.413	0.063	-0.05 (0.037), 0.12	**0.048**
24,25(OH)_2_D	25(OH)D		-0.00085 (0.03), 0.81	-0.001 (0.003), 0.76	0.56	0.112 (0.006), **<0.001**	0.290
FePi%	PTH		0.035 (0.019), **0.07**	0.022 (0.024), 0.28	0.54	0.022 (0.024), 0.28	0.54
FePi%	PTH	1,25(OH)2D	0.035 (0.019), **0.052**	0.027 (0.028), 0.336	0.50	0.027 (0.024), 0.26	0.50
FePi%	PTH	1,25(OH)2D, FGF23	0.031 (0.019), 0.09	0.028 (0.029), 0.35	0.51	0.028 (0.024), 0.25	0.51
FePi%	1,25(OH)2D		-0.026 (0.021), 0.2941	-0.09 (0.04), 0.06	0.21	-0.14 (0.04), **0.0009**	**0.017**
FePi%	1,25(OH)2D	PTH	-0.03 (0.024), 0.2028	-0.009 (0.04), **0.048**	0.19	-0.108 (0.04), **0.0007**	**0.0204**
FePi%	1,25(OH)2D	FGF23, PTH	-0.026 (0.025), 0.55	-0.09 (0.05), **0.052**	0.129	-0.108 (0.04), **0.0045**	**0.018**
FePi%	1,25(OH)2D	FGF23, PTH, SCa	-0.015 (0.026), 0.49	-0.08(0.04), 0.060	0.13	-0.108(0.04), **0.0046**	**0.018**
FePi %	FGF23		0.03 (0.01), **0.0092**	0.009 (0.027), 0.724	0.1362	0.004 (0.0011), 0.8112	0.1362
FePi %	FGF23	PTH	0.035 (0.01), **0.014**	0.007 (0.02), 0.78	0.136	0.0035 (0.01), 0.84	0.136
FePi %	FGF23	PTH, 1,25(OH)2D	0.033 (0.01), **0.029**	-0.003 (0.03), 0.901	0.237	-0.002 (0.01), 0.906	0.237
FECa%	PTH		-0.008 (0.004), 0.065	-0.008 (0.005), 0.13	0.76	-0.011 (0.005), **0.052**	0.603
FECa%	PTH	1,25(OH)2D	-0.008 (0.004), 0.058	-0.010 (0.004), 0.058	0.591	-0.010 (0.004), 0.058	0.591
FECa%	1,25(OH)2D	PTH	0.003 (0.005), 0.536	-0.01 (0.007), 0.168	0.188	-0.01 (0.007), 0.168	0.188

We compared serum variables in normocalciuric (defined as U Ca <200 mg per 24 hours in females and <250 mg per 24 h in males) or hypercalciuric (defined as U Ca >200 mg per 24 hours in females and >250 mg per 24 h in males) male and female subjects in the TSF group. We found no differences in any of the analytes (Table 3).

**Table 3:** Urine Ca and serum analytes in male and female normocalciuric SF (NC; males, U Ca <250 mg/ 24 h; females U Ca <200 mg/24 h) or hypercalciuric SF (HC; male U Ca >250 mg/24 h; female U Ca > 200 mg/24 h); mean +/- SEM.

Analyte, urine (U) or Serum (S)	NC male SF (N = 45)	HC male SF (N = 32)	P value; male NC vs. HC SF	NC female SF (N = 42)	HC female SF (N = 30)	P value; female NC vs. HC SF
**U Ca, mg/24 h**	152.2 ± 9.0	410.6 ± 26.5	<0.0001	123.2 ± 6.4	282.9 ± 14.4	<0.0001
**S Ca, mg/dL**	9.3 ± 0.1	9.4 ± 0.1	0.772	9.3 ± 0.1	9.5 ± 0.1	0.388
**S P, mg/dL**	3.3 ± 0.07	3.1 ± 0.10	0.117	3.6 ± 0.09	3.5 ± 0.10	0.607
**S 1,25(OH)**_**2**_ **D, pg/mL**	41.4 ± 1.7	41.1 ± 2.1	0.914	46.4 ± 1.79	46.3 ± 3.25	0.451
**S 24,25(OH)**_**2**_ **D**	3.0 ± 0.2	2.8 ± 0.2	0.869	3.4 ± 0.2	3.3 ± 0.4	0.275
**S FGF-23, pg/mL**	60.4 ± 2.7	65.1 ± 9.0	0.403	61.3 ± 3.2	63.2 ± 3.6	0.855

Food intake based on Food Frequency Questionnaires in the CSF, TSF and control groups is presented **in** Table 4. Of note, data was not available in all patients. There were no differences between the intake of calories, protein, animal protein, sodium, calcium, phosphorus, magnesium, potassium, and vitamin D between the various groups.

**Table 4:** Dietary intake variables in calcium stone-formers and controls. All values are represented as mean ± standard error. CSF = confirmed; TSF = total (confirmed plus presumed calcium SF).

Variable	Controls (N = 195)*	Confirmed calcium stone-formers, CSF (N = 89)*	P	Total stone-formers, TSF (N = 144)*	P
**Calories, kcal**	2341.9 ± 77.7	2300.79 ± 118.4	0.771	2311.3 ± 97.9	0.807
**Fat, g**	87.72 ± 3.6	88.57 ± 5.9	0.902	88.31 ± 4.5	0.919
**Carbohydrates, g**	283.22 ± 9.2	276.79 ± 13.6	0.696	279.71 ± 11.3	0.810
**Total Protein, g**	97.65 ± 3.4	97.37 ± 5.2	0.964	97.03 ± 4.5	0.914
**Animal Protein, g**	65.8 ± 2.6	68.25 ± 4.1	0.615	67.14 ± 3.4	0.752
**Plant Protein, g**	31.68 ± 1.2	28.92 ± 1.6	0.182	29.69 ± 1.5	0.306
**Alcohol, g**	10.9 ± 1.14	7.51 ± 1.6	0.082	7.79 ± 1.2	0.065
**Sodium, mg**	3763.6 ± 132.1	3665.25 ± 218.9	0.701	3710.03 ± 182.1	0.812
**Calcium, mg**	1403.50 ± 51.8	1306.5 ± 81.4	0.316	1309.75 ± 69.15	0.280
**Phosphorus, mg**	1770.3 ± 58.94	1699.8 ± 89.13	0.510	1697 ± 77.3	0.452
**Magnesium, mg**	385.9±11.97	370.0±19.23	0.491	364.9±16.34	0.299
**Potassium, mg**	3597.12±108.9	3421.2±168.1	0.381	3363.9±145.2	0.200
**Vitamin D, IU**	308 ± 14	293 ± 22	0.584	285 ± 18	0.351

* FFQ were not available on all subjects.

In the control population, 20% of subjects reported a family history of kidney stones, 74% of subjects reported no family history of stone disease, and 6% were unaware of a history of kidney stones. In contrast, 42% of TSF had a family history of kidney stone disease, 55% did not, and 3.2% of patients were unaware of a history of kidney stones. Thiazide use was similar in the CSF (7.5%), TSF(8.7%) and control (11%) groups.

## Discussion

Previous studies have reported elevated or high normal serum concentrations of 1,25(OH)_2_D, the active metabolite of vitamin D, among patients referred to specialty clinics with urinary stone disease and hyperparathyroidism, absorptive hypercalciuria or renal hypercalciuria [13–17]. However, systemic differences in Ca and vitamin D metabolism have not been previously investigated in a community-based population of first-time male and female stone-formers. Likewise, urinary excretion of solutes has not been assessed in the same cohort of patients. The current study reveals higher serum Ca and 1,25(OH)_2_D concentrations even among these first-time Ca stone-formers relative to matched controls. Similar conclusions can be drawn using data in the current study obtained from patients with confirmed Ca containing stones or from the expanded cohort of first time stone formers in whom Ca stones are also likely to be present. The data indicate that the increase in serum 1,25(OH)_2_D among first-time stone-formers could be related to reduced activity of the 24-hydroxylase enzyme (measured as the ratio of serum 24,25(OH)_2_D to 25(OH)D) that normally metabolizes both 1,25(OH)_2_D to a less active metabolite, 1,24,25-trihydroxyvitamin D.

In our study, first time stone-formers had higher serum Ca and 1,25(OH)_2_D levels than the non-stone forming controls, suggesting that first-time Ca stone formation is a manifestation of altered Ca and vitamin D regulation. Increased serum 1, 25(OH)_2_D concentrations would predispose stone-formers to intermittent hypercalciuria following ingestion of increased amounts of dietary Ca. The higher 1,25(OH)_2_D levels in the Ca stone-formers might also predispose them to increased urinary supersaturation under other environmental influences, e.g. dehydration, vitamin supplementation, or sunlight exposure. Our data suggest that further investigations of known regulators of Ca and vitamin D metabolism will help in the medical management of the first time stone formers and prevent stone recurrence through dietary modifications and pharmaceutical interventions.

We and others have shown that patients with familial adult and infantile hypercalcemia, have hypercalciuria and nephrolithiasis that are associated with mutations of the *CYP24A1* gene [19, 20, 24, 26–28]. Genome-wide association studies have shown that the *CYP24A1* gene is associated with serum calcium [25] and genomic variants of the *CYP24A1* gene have been identified in a small number of calcium stone formers to date [34]. In a cohort of patients with kidney stones, high normal serum Ca and low normal PTH concentrations, nine known sequence variants of the *CYP24A1* gene were detected [34]. In a second cohort with kidney stones and a tendency towards hypercalciuria, seven known sequence variants, and a single novel heterozygous non-synonymous sequence change together with a homozygous synonymous change have been detected [34]. It is not known if these sequence variants are associated with reduced 24-hydroxylase activity since vitamin D metabolite concentrations were not measured.

Serum PTH concentrations were similar in the two groups, and thus do not appear responsible for increased 25-hydroxyvitamin D-1α-hydroxylase (Cyp27B1) [35, 36] activity and increased 1,25(OH)_2_D synthesis. Reduced inhibition of 1,25(OH)_2_D synthesis by FGF-23 [37] could potentially be responsible for the elevation in serum 1,25(OH)_2_D concentrations, despite identical FGF-23 concentrations in stone-formers, since the expected significant inverse correlation between FGF-23 and 1,25(OH)_2_D_3_ observed in control subjects (P<0.001) was not observed in Ca kidney stone-formers (P = 0.16).

Of note, in the current study there is a trend towards higher PTH concentrations in the stone-formers (P = 0.096) and PTH levels in the stone-formers are higher despite higher serum 1,25(OH)_2_D and Ca concentrations. The lack of inhibition of serum PTH concentrations by increased serum 1,25(OH)_2_D and Ca concentrations in stone formers suggests the presence subtle alterations of the parathyroid gland to inhibitory signals in stone formers. Recent genome wide association studies have implicated up to six loci in the regulation of serum Ca [25]. In addition to *CYP24A1*, discussed above, implicated genes include the Ca sensing receptor, diacylglycerol kinase, and GATA transcription factor genes, all of which may be involved in parathyroid gland signaling mechanisms. Whether sequence variants or mutations in these or other genes are present our cohort of stone-formers has not been assessed. It is unclear if other genes identified in the recent meta-analysis such as *CARS*, the causative gene for the Beckwith-Weidemann syndrome, and *GCKR* might be associated with altered Ca and 1,25(OH)_2_D signaling in the parathyroids [25].

Despite higher serum calcium, the SF and control groups had similar urinary calcium excretions. Any explanation is speculative, but might relate to the fact these are first time and not recurrent SF. There were also no differences amongst groups in the intake of calcium and vitamin D. Further analysis of serum analyte concentrations based on the presence or absence of hypercalciuria in male and female stone formers revealed no differences in normocalciuric versus the hypercalciuric groups.

A recent report of 356 male incident stone formers compared plasma concentrations of 25-hydroxyvitamin D, 24,25-dihydroxyvitamin D, parathyroid hormone, fibroblast growth factor 23, calcium, phosphate, and creatinine with those found in control subjects [18]. Of note, this study was limited to male subjects. Small and statistically non-significant differences in 1,25(OH)_2_D and FGF23 levels were found in cases and controls. However, after adjusting for multiple covariates, the odds ratios of incident symptomatic kidney stones in the highest compared with lowest quartiles were 1.73 (P for trend 0.01) for 1,25(OH)2D and 1.45 (P for trend 0.03) for FGF23. Unlike our study, Taylor *et al*. [18] did not observe or comment upon differences in the ratio of serum 24,25(OH)_2_D to 25(OH)D in stone formers compared to controls. Furthermore, we found differences in the relationship between FGF-23 and urinary phospate excretion and 1,25(OH)_2_D amongst stone-formers and controls suggesting alterations in the responsiveness of the nephron to FGF 23 in stone formers. Urinary solute concentrations were not examined in the Taylor study.

The limitations of this study include that the cohort was limited to a Caucasian population in the upper midwestern United States, the effect of Ca or vitamin D loading was not determined in either group, and the relationships of the hormonal alterations to urinary calcium were not assessed. Not all patients had an analysis of stones. Furthermore, sequencing of genes involved in the production and degradation of 1,25(OH)_2_D was not performed.

## Conclusions

Systematic study of a community- based cohort of first-time stone-formers revealed elevated serum Ca, 1,25(OH)_2_D, and PTH concentrations. There was evidence for diminished 24-hydroxylase (Cyp24A1) activity which could contribute to the increased 1,25(OH)_2_D concentrations observed in these stone-forming subjects. Furthermore, an impaired inhibitory response of the 25-hydroxyvitamin D 1-hydroxylase (Cyp27B1) to circulating FGF-23 in stone-formers could also contribute to the higher 1,25(OH)_2_D concentrations. Our study demonstrates that altered Ca and vitamin D metabolism were common in a relatively large cohort of first-time Ca stone-formers.
